# Receptor Tyrosine Kinase (RTK) Mediated Tyrosine Phosphor-Proteome from Drosophila S2 (ErbB1) Cells Reveals Novel Signaling Networks

**DOI:** 10.1371/journal.pone.0002877

**Published:** 2008-08-06

**Authors:** Srinivasan Krishnamoorthy

**Affiliations:** Pediatric Surgical Research Laboratories, Department of Surgery, Massachusetts General Hospital, Harvard Medical School, Boston, Massachusetts, United States of America; Baylor College of Medicine, United States of America

## Abstract

Protein phosphorylation mediates many critical cellular responses and is essential for many biological functions during development. About one-third of cellular proteins are phosphorylated, representing the phosphor-proteome, and phosphorylation can alter a protein's function, activity, localization and stability. Tyrosine phosphorylation events mediated by aberrant activation of Receptor Tyrosine Kinase (RTK) pathways have been proven to be involved in the development of several diseases including cancer. To understand the systems biology of RTK activation, we have developed a phosphor-proteome focused on tyrosine phosphorylation events under insulin and EGF signaling pathways using the PhosphoScan® technique coupled with high-throughput mass spectrometry analysis. Comparative proteomic analyses of all these tyrosine phosphorylation events revealed that around 70% of these pY events are conserved in human orthologs and paralogs. A careful analysis of published *in vivo* tyrosine phosphorylation events from literature and patents revealed that around 38% of pY events from Drosophila proteins conserved on 185 human proteins are confirmed *in vivo* tyrosine phosphorylation events. Hence the data are validated partially based on available reports, and the credibility of the remaining 62% of novel conserved sites that are unpublished so far is very high but requires further follow-up studies. The novel pY events found in this study that are conserved on human proteins could potentially lead to the discovery of drug targets and biomarkers for the detection of various cancers and neurodegenerative diseases.

## Introduction

Unraveling signaling networks from the perspective of understanding systems biology has been the most popular approach to set up an effective platform to identify sensitive cell signaling nodes leading to novel drug targets [1]. High-throughput mass spectrometry approaches along with improved techniques such as SILAC for quantitative proteomics have provided the building blocks of the current knowledge base for this new grammar of drug discovery [2]. About 60% of Drosophila proteins have human homologues with well-conserved canonical signaling cascades. Because Drosophila is a less complex model system than a vertebrate, it gives an opportunity to analyze complex signaling networks and translate the findings to identify novel drug targets for human diseases. Datasets from model systems with conserved canonical signaling pathways (such as Drosophila) play an important part in rapidly generating a knowledge base.

Aberrant activation of RTK pathways has been shown to be involved in the development of various types of cancers [3]–[6]. Recent therapeutic approaches have involved the development of drugs in the form of small molecules or monoclonal antibodies that block or control activation of tyrosine phosphorylation events on specific proteins to control the progression of cancer; some of these are available currently in the market [7],[8].

The technically challenging nature of tyrosine phosphorylation modifications is mainly attributed to: 1) occurrence of tyrosine phosphorylation modifications on very low-abundance proteins, 2) lower relative abundance of tyrosine phosphorylations compared to serine and threonine phosphorylations, 3) very low stoichiometry and 4) labile nature of pY events during various chemical manipulations as required for mass spectrometry analysis [2]. Unlike serine and threonine phosphorylation modifications, the rules of consensus do not work well with tyrosine phosphorylation, and programs based on algorithms to predict tyrosine phosphorylation have not matched experimental outcomes. Hence a comprehensive high-throughput effort focused on generating tyrosine phosphorylation profiles will add to the knowledge base used to construct robust algorithms based on large datasets.

Here we report a phosphor-proteome from Drosophila exclusively focused on tyrosine phosphorylation events under insulin and EGF signaling pathways. We also present the salient features of the Drosophila proteome architecture and the comparative proteomic analysis for conserved tyrosine phosphorylation events on human proteins.

## Results

### Phosphopeptide profiles from Drosophila S2 (EGFR) cells

Using the PhosphoScan® technique [9], a high-throughput mass spectrometric analysis of lysates after independent stimulation of the cloned human EGF receptor (ErbB1) and the endogenous insulin-like receptor (InR) in S2 cells yielded a tyrosine phosphopeptide spectrum of 658 tyrosine phosphorylated peptides. Out of these 658 phosphopeptides, 511 were non-redundant consisting of 543 individual tyrosine phosphorylation (pY) events from 290 different proteins spanning the entire Drosophila proteome, with 1-14 sites per protein. The time points of RTK activation (0, 2, 8 and 12 minutes) used for mass spectrometry analysis represent the dynamics of D-ERK activation upon RTK stimulation. RTK-mediated activation of D-ERK in these S2 (EGFR) cells is initiated as early as 2 minutes and decreases after 12 minutes.

**Figure 1:**
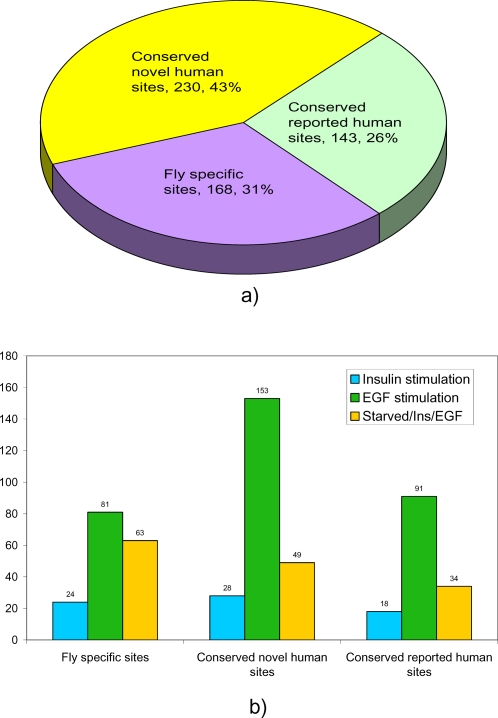
Classification of RTK-mediated pY proteome based on (a) organ systems, (b) cellular processes and (c) signaling pathways.

Activation of the endogenous insulin receptor yielded 63 peptides with 70 pY events on 38 different proteins whereas activation of over-expressed ErbB1 in S2 cells yielded 283 phosphopeptides containing 325 pY events on 177 proteins. About 20% (116/511) of phosphopeptides containing 146 pY events on 79 proteins were also found in serum-starved samples that were devoid of GF treatment (Figure 1).

We found 6 pY events on Drosophila Insulin-like receptor (InR) out of which three are conserved in the human insulin receptor and IGF-1R. We identified three phosphorylation sites (Y1110, Y1172 and Y1192) on the cloned ErbB1 receptor in the S2 cell line used in this study.

### Architecture of RTK-mediated Tyrosine phosphor-proteome

Classification of phosphoproteins revealed various functional categories such as kinases, signaling adaptors, actin binders, microtubule regulators, cell-adhesion molecules, GEFs/GAPs, ubiquitin modifiers, transcriptional and translational regulators, intracellular transport factors, endocytotic epsins, molecular chaperones and proteins involved in various biosynthetic pathways including carbohydrate metabolism. A detailed classification of Drosophila tyrosine phosphopeptides into 16 different categories is given in Table S1 along with information about activated RTK (InR or ErbB1) corresponding to each peptide.

The phosphopeptide profiles obtained upon activation of insulin and EGF RTKs gave the largest spectrum of pY events, representing all major signaling pathways (MAPK pathway, PI3Kinase pathway, STAT pathway and PLCγ pathway) under RTK activation responsible for cell growth, proliferation, survival and differentiation.

About one-fourth of the phosphoproteins (74/290) found in this study have no known molecular or biological function, but about half of these have motifs suggestive of their molecular function.

**Figure 2:**
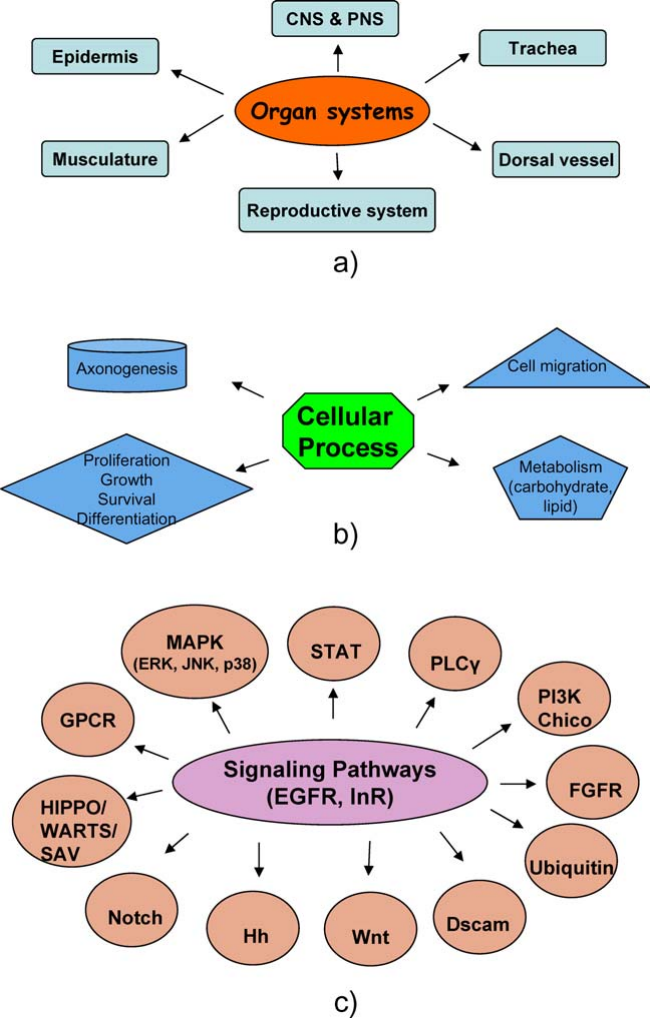
Overall comparative proteomic analysis of Drosophila and human proteomes. (a) analysis shows a total of 541 pY events, of which 373 are conserved in human proteins and 168 are fly specific. (b) The bar chart represents the proportion of pY events specific for insulin and EGF RTKs.

In the context of Drosophila development, phosphoproteins found in this study are involved in early embryogenesis, cellularization, early and late blastulation, gastrulation, patterning and cell migration. Proteins involved in major organogenesis pathways such as heart and muscle development, tubulogenesis (tracheal and myotube development), dorsal vessel, CNS development and reproductive system and down-regulation of RTKs (receptor endocytosis pathway) are also phosphorylated. A detailed illustration of various organ systems, cellular processes and signaling pathways represented by the tyrosine phosphor-proteome is illustrated in Figure 2.

In the category of RTKs other than insulin and EGFR, the *pvr*-(PGDF and VEGF homologue) gene is a major protein with 13 pY events. Classification of pY events based on cellular biological processes yielded four major categories: 1) signaling proteins in CNS development (101 pY events on 44 proteins), 2) dorsal closure (65 pY events on 11 proteins), 3) cell polarity (29 pY events on 19 proteins) and 4) Ras-interacting proteins that included REF and GEFs (22 pY events on 18 proteins).

### Comparative analysis of Drosophila phosphoproteome against human protein database

Using InParanoid (SBC) and HomoloGene (NCBI) browsers and the eukaryotic ortholog list from Flybase, a search for human orthologs of Drosophila proteins yielded 216 protein matches. For those cases in which we could not identify any human ortholog, we conducted two different BLAST searches (BLAST2 and BLASTP) to find out the nearest human protein match. ClustalW2 (EMBL-EBI) analysis of 511 tyrosine phosphopeptides on 290 proteins was done using human protein databases (Swiss-Prot and NCBI) to uncover all the pY events conserved in respective human homologous proteins. The standard parameters (Protein Gap open penalty: 10.0; Gap Extension penalty: 0.2; Protein matrix: Gonnet; Protein ENDGAP: -1 and GAPDIST: 4) were used for the entire analysis. Out of these, 175 human protein matches had conserved phosphorylated tyrosines. Often a single Drosophila protein match contained more than one human protein with conserved tyrosine.

About 75.1% (244/325) of pY events upon ErbB1 stimulation are conserved in the human proteome compared to 65.7% (46/70) from activation of insulin receptor and 56.8% (83/146) of pY events common to both EGF and insulin stimulation. A total of 373 pY events (68. 9%) in this data are conserved in the human proteome (Figure 1).

The human proteins with conserved tyrosines had protein homology ranging from as high as 95% to as low as 20%. We found many tyrosines conserved on the human tyrosine kinases, receptors and a variety of cytoplasmic and membrane-bound proteins involved in CNS development (especially the process of axon guidance and retinal degeneration), cell polarity and carbohydrate metabolism. We checked the various literatures (PubMed) and databases (PhosphoSite®) hosted by CST Inc, UniProtKB/Swiss-Prot (http://www.expasy.org/uniprot) and PhosphoPep (http://www.phosphopep.org/) to see if the conserved tyrosines are actual *in vivo* sites on human proteins. Based on the available literature, 38% of the hits found in this study are already found and reported as confirmed *in vivo* tyrosine phosphorylation sites in human proteins. Table 1 contains a category-wise count of conserved pY events that are confirmed *in vivo* sites in human proteins as well the novel sites that are not reported so far.

**Table 1:** Summary of proportion of tyrosine phosphorylation sites in Drosophila RTK proteome based on molecular functional categories.

Major Group	Molecular Classification	Fly specific	Conserved pY events reported in literature to date		Conserved novel pY events not yet reported	
			# of pY events	# of proteins	# of pY events	# of proteins
1	Cytoplasmic tyrosine kinases	12	10	7	8	5
2	Receptor Tyrosine kinases	7	7	3	11	4
3	Cytplasmic S/T kinases	4	12	11	4	4
4	Receptor S/T kinases	-	3	2	3	2
5	Other kinases	9	1	1	7	4
6	Other receptors	13	15	10	26	13
7	Ligands for RTKs	1	-	-	-	-
8a	Signaling adaptor proteins	9	16	11	8	7
8b	Actin binding proteins	16	14	5	10	7
8c	Other proteins in cytoskeleton	5	4	3	6	4
8d	Cell adhesion proteins	-	1	1	5	4
9	Ras interacting proteins	8	6	5	11	11
10	Proteases	5	3	3	10	8
11a	Cation transporters	-	1	1	6	7
11b	Signaling related transporters (ABC family)	1	3	3	4	3
11c	Vesicle mediated transporters	3	4	4	4	4
11d	Oligopeptide transporters	3	-	-	-	-
11e	Nucleotide transporters	1	-	-	2	1
12	Carbohydrate metabolism related transporters	7	3	3	10	7
13	Ion channel proteins	4	-	-	2	2
14	Molecular chaperones	-	2	2	1	1
15	Transcription related	3	1	1	5	4
16	Translation related	2	4	4	7	6
17	Other signaling proteins	14	7	6	27	17
18	Proteins with known motifs & unknown functions	13	11	9	21	22
19	Proteins with unknown motifs & functions	28	15	5	32	28
Total		168	143	100	230	175

**Figure 3:**
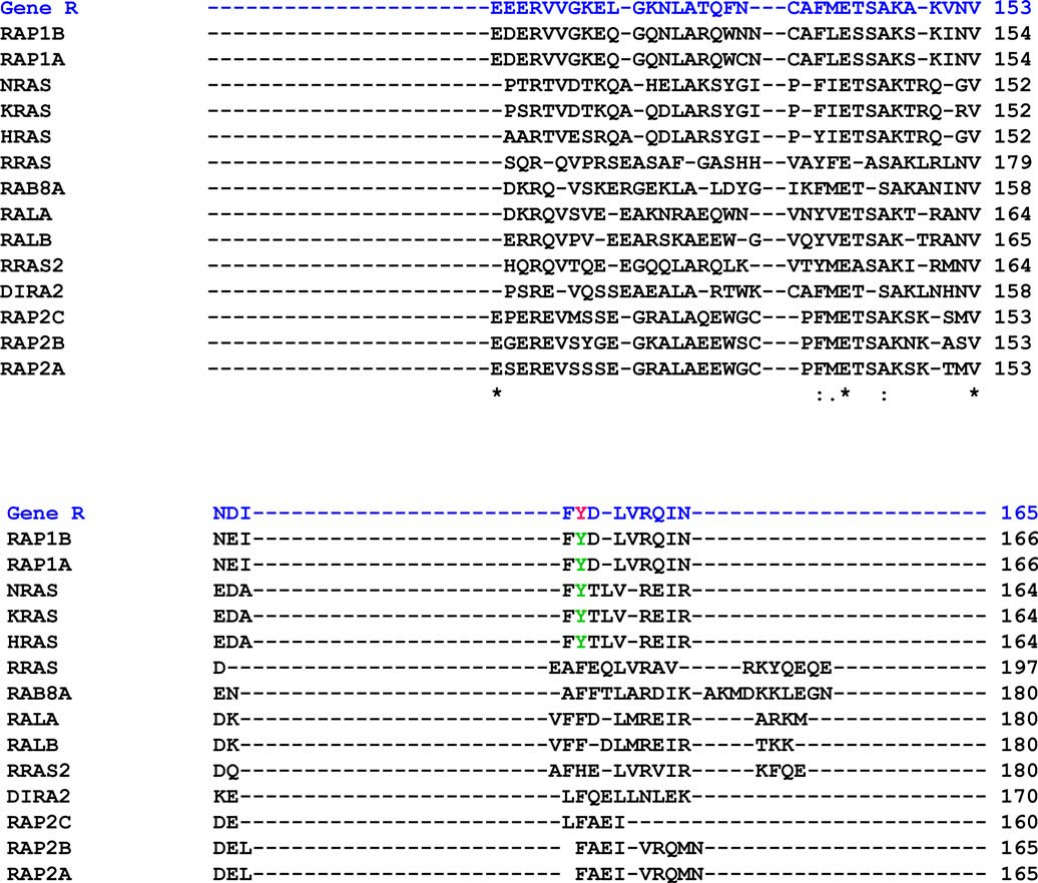
Tyrosine phosphorylation in Roughened (Gene R) from Drosophila is conserved in human H-RAS, K-RAS, N-RAS and R-RAS. The Drosophila protein sequence (blue with the phosphorylated tyrosine in red) aligns with several human protein sequences (black). The conserved tyrosines in the human proteins are highlighted in green.

Phosphorylated tyrosines on Drosophila are conserved on more than one human protein with equivalent molecular function even if they are distantly related, irrespective of the extent of protein homology. For example, the tyrosines Y1545 and Y1550 on Drosophila insulin receptor (InR) are conserved in human INR, IGF-1R RET oncogene, LTK, MUSK, ALK (anaplastic lymphoma kinase), ALK Ki-1 variant, TFG/ALK fusion kinase, NTRK2, NTRK2 and MERTK. From the same perspective we find a conserved pY event on the Ras interacting protein coded by *roughened* (R) which is conserved in H-Ras, K-Ras R-Ras, N-Ras, RAP1A and RAP1B. A detailed clustal map showing the clustal alignment of Gene R with human proteins showing conserved tyrosines is given in Figure 3.

### Many signaling proteins with conserved tyrosines are involved in disease phenotypes

Tyrosine phosphorylation events conserved on proteins involved in muscular dystrophy, retinal degeneration, Alzheimer's, breast cancers, acute myelogenous leukemia (AML), aggressive melanomas and several other diseases are very interesting molecular targets warranting further validation in transgenic mouse models and tumor cell lines. In total we find 30 pY events on 25 proteins involved in various types of cancers, 8 pY events on 8 leukemia related proteins, 34 pY events on 30 proteins involved in various genetic disorders, and 18 pY events on 14 proteins involved in various neurodegenerative diseases. A list of important proteins involved in human disease development with the location of conserved pY residues is given in Table 2 and a detailed list of all the human proteins with conserved tyrosines that are phosphorylated in respective Drosohila proteins is given in Tables S2 and S3. A few of these conserved pY events are specific to a particular isoform of the respective protein orthologs. A small number of pY events on fly proteins reveal natural (Y-F, Y-H, Y-W and Y-D) substitutions in the respective human orthologs (Table S2).

**Table 2:** Selected list of important human proteins with conserved fly yrosine phosphorylation involved in disease phenotypes.

Protein with putative pY event	Accession #	Location of pY	Disease
**ABCG1:Y84**	P45844	Cytoplasmic domain	Over-expressed in patients with Tangier disease
**ABCG2:Y44**	Q9UNQ0	unknown	Up-regulated in brain tumors.
**ADAMTS20:Y 1572**	P59510	TSP type-1 13	Over-expressed in several brain, colon and breast carcinomas.
**ATP1A3:Y532**	P13637	Cytoplasmic domain	Defects in ATP1A3 are the cause of dystonia-12 (DYT12)
**BIRC6:Y4102, Y4130**	Q9NR09	unknown	Expressed in brain cancer cells.
**CACNA1F:Y1769**	O60840	Cytoplasmic domain	Congenital stationary night blindness type 2 (CSNB2)
**DMD-Dp140bc:Y934**	NP_004014	unknown	DMD are the cause of Duchenne muscular dystrophy (DMD), Becker muscular dystrophy (BMD), X-linked dilated cardiomyopathy (XLCM)
**ETV6:Y391**	P41212	ETS domain	Various leukemias like AML, CMML, ALL, MDS etc.
**Fibulin6:Y5045; Y5296**	Q96RW7	Nidogen G2 beta-barrel domain	Age-related macular degeneration type 1 (ARMD1)
**GAS7:Y31**	O60861	WW domain	A chromosomal aberration involving GAS7 is a cause of acute myeloid leukemia
**HMCN1:Y 2861**	Q96RW7	Ig-like C2-type 26 between C2-type 28	Defects in HMCN1 are a cause of age-related macular degeneration type 1 (ARMD1)
**HMCN1:Y3053**			
**INVS:Y566**	Q9Y283	IQ 1 domain	Defects in INVS are the cause of nephronophthisis 2 (NPHP2)-Infant kidney disease
**MXRA5:Y2709**	Q9NR99	Ig-like C2-type 11	Expressed in arthritic tissues, Over-expressed in centenarians. Expression is reduced from young to old but increased from old to centenarians.
**MXRA5:Y2717**			
**PROM1:Y828**	O43490	Cytoplasmic domain	Defects in PROM1 are the cause of an autosomal recessive form of retinal degeneration.
**HRAS:Y157**	P01112	Unknown	Mutated in various cancers
**KRAS:Y157**	P01116	Unknown	
**NRAS:Y157**	P01111	unknown	
**REG1A:Y48**	P05451	C-type lectin domain on lithostathine 1 alpha chain	Alzheimer disease and Down syndrome patients show enhanced accumulation of PSP-like proteins in their brains
**REG1A:Y49**			
**SPAST:Y149**	Q9UBP0	MIT domain	Defects in SPAST are the cause of spastic paraplegia type 4 (SPG4)
**TNKS2:Y569**	Q9H2K2	ANK 11 repeat	Highly expressed in mammary gland, breast and breast carcinoma
**UNC5C:Y449**	O95185	Cytoplasmic domain	Down-regulated in multiple cancers including colorectal, breast, ovary, uterus, stomach, lung, or kidney cancers
**VEGFR3:1063**	P35916	Cytoplasmic domain	Juvenile hemangioma

## Discussion

The tyrosine phosphopeptide profiles presented here represent the largest dataset reported in Drosophila to date. This dataset is unique because it highlights activated proteins upon activation of growth factor RTKs (endogenous insulin RTK and human EGFR). Many of the novel phosphorylation events found in this study on proteins previously not known to be involved in RTK pathways, represent new signaling nodes that merit further validation.

One of the major issues in the conventional SDS-PAGE method to identify pY modifications is the significant loss during recovery of peptides after in-gel digestion of total protein entrapped in the PAGE gel matrix. Even though this is not an issue in the case of protein identification as such, it may pose many technical impediments for identifying post-translational modifications, especially on tyrosine (pY), which are labile during chemical processing of peptides and recovery from the gel matrix. The PhosphoScan® technique is a non-gel based method involving direct immunoaffinity precipitation of pY peptides concentrated from whole-cell-lysate digests; it facilitates identification of pY sites on both less-abundant proteins and proteins of low stoichiometry of phosphorylation, and importantly, avoids technical complications of peptide recovery from gel matrix. The quality and quantity of phosphopeptides obtained in this study is far better than methods using various IMAC columns. Evaluating various enrichment techniques for tyrosine identification of tyrosine phosphorylations, Schumacher et al [10] found that immunoaffinity precipitation is superior to the immunoaffinity chromatography method.

Peptides containing multiple acidic residues (D and E) around phosphorylated tyrosines are technically difficult for mass spectrometric analysis. Very low stoichiometry of tyrosine phosphorylation makes it almost impossible to detect many tyrosine modifications on low-abundance proteins. A typical example in our case was the identification of tyrosine phosphorylation sites on a nuclear import protein (Dim7) with a very low stoichiometry (0.004 moles/mole). High cell quantities (2×10^9^ cells) used in conventional SDS-PAGE methods failed to identify any pY sites on this protein. But using the PhosphoScan® technique, we identified two pY sites on this protein, with just 1×10^8^ cells [S. Krishnamoorthy and L.A. Perkins, unpublished].

### Overlap of Drosophila phosphoproteome data with other HT screens

A minimal overlap of pY sites from Kc cell proteome data is seen with respect to tyrosine phosphorylation [11]. A considerable overlap of proteins representing various phosphopeptides from our HT mass spectrometry data from insulin stimulation was seen with the candidate gene list from RNAi screen data for insulin RTK mediated ERK activation [12]. But the EGF RTK mediated *HeLa* cell phosphor-proteome data showed overlap of only one pY site with respect to tyrosine phosphorylation modifications, even though many candidate proteins with conserved tyrosines from our data are either serine or threonine phosphorylated upon EGFR activation [2]. A recent report [13] on signaling networks assembled by oncogenic EGFR and c-Met pathways and a HT mass spectrometry analysis of pervanadate-stimulated S2 cells [14] data have a considerable overlap with our data.

**Figure 4:**
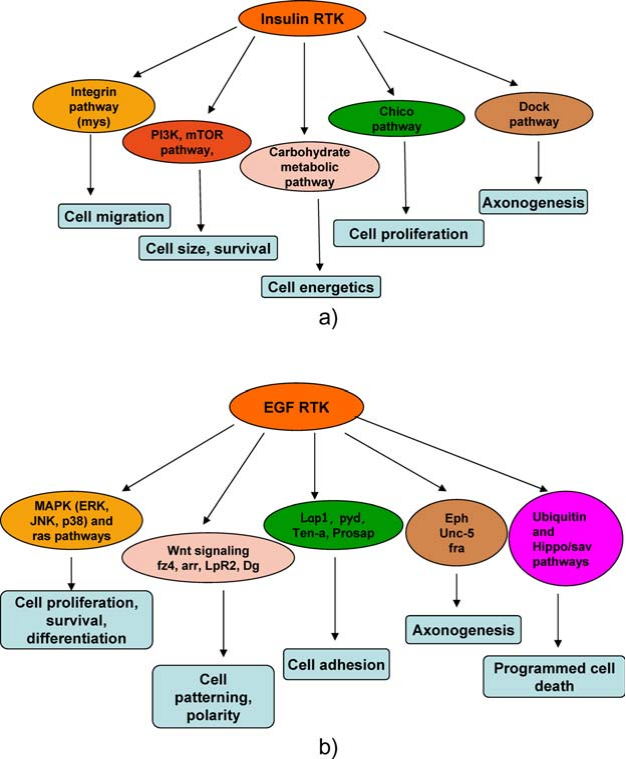
Tyrosine phosphorylation signaling pathways in Drosophila. a) Insulin RTK mediated tyrosine signaling pathways. b) EGF RTK mediated tyrosine phosphorylation signaling pathways.

The RTK mediated tyrosine phosphor-proteome network under insulin and EGF RTKs has not only validated existing tyrosine phosphorylated signaling nodes but also reveals several novel insights in to regulation of RTK signaling and crosstalk with various other pathways (Figures 4a and 4b).

### Translational value of conserved pY events in Drosophila proteome and future directions

The novel tyrosine phosphorylation events from the Drosophila proteome conserved in 100 human orthologs and paralogs constitute a valuable resource to translate the missing signaling connections and nodal points in the human proteome from the perspective of disease development. The data warrants further validation in human tumor cell lines and tissue samples to see if these pY events are up-regulated or down-regulated in GF signaling with respect to human disease phenotypes.

Recent reports indicate the importance of genes in glycolytic pathways in cancer progression [15]. Mechanisms of tumor growth based on the selective switching of cellular processes towards anabolic pathways rather than oxidative phosphorylation also stress the importance of glycolytic proteins in cancer development [16]. Our study in the fly proteome reveals that several proteins involved in glycolytic pathways are tyrosine phosphorylated and that these candidate human proteins with conserved tyrosine residues merit further study. Upregulation of monocarboxylic transporters (MCA) in Type-1 diabetic patients indicate the possibility of increased capacity of the brain to use non-glucose substrates to meet energy requirements during hypoglycemia [17]. Our study reveals that several MCA transporters are tyrosine phosphorylated upon RTK activation. It will be interesting to see if the human orthologs with conserved phosphorylated tyrosines found in this study are involved in similar mechanisms in brain-cell energetics.

Based on the available protein expression data, interesting candidate proteins could be selected for analysis of the dynamics of tyrosine phosphorylation with respect to disease development. Reverse-phase protein microarrays could be a very useful tool in this direction [18]. Peptide arrays containing novel conserved pY modifications could be used to probe SH2 domain containing protein arrays to expand the signaling network with respect to a particular pathway [19].

## Materials and Methods

### Cell culture

S2 cells stably transfected with human ErbB1 (gift from David Baker, Leiden University Medical Center, Netherlands) were maintained in Schneider's Drosophila medium (CAMBREX Inc.) supplemented with 10% fetal bovine serum (Mediatech, Inc.), penicillin and streptomycin.

### Growth factor stimulation

S2 (EGFR) cells were grown in 15-cm culture dishes to about 80% confluency. The cells were starved by replacing complete medium with minimal medium without serum (15mL of minimal medium per plate) for about 18–20 hours. The next day cells were stimulated with EGF (100ng/mL of medium) or insulin (5 ug/mL of medium) for 2, 8 and 12 minute time intervals. A total of 10 plates that gave approximately 2×10^8^ cells were used per stimulation. A set of 10 plates of overnight-starved cells was used as the control. The cells were lysed after stipulated time of growth factor treatment using the lysis buffer for containing 6M urea. As per the manufacturer's directions, 10 mL of lysis buffer were used to lyse cells from a batch of ten 15-cm culture plates. The lysate from insulin and EGF treated samples were subjected to further processing as per the manufacturer's directions (PhosphoScan® Kit (P-Tyr-100) #7900, Cell Signaling Technology Inc., Danvers MA USA).

### Peptide extraction, LC-MS/MS analysis and assignment of tyrosine phosphorylation

Digestion of total lysate with trypsin, reverse phase solid phase extraction of digests, immunoaffinity purification of phosphopeptides, analysis of phosphopeptides by LC-MS/MS analysis, evaluation of MS/MS spectra using Sequest browser and assigning phosphopeptide sequences and review of assigned peptide sequences using a two step process were done at the Cell Signaling Technology facility following the methods described by Rush et al [9].

### Identification of human homologues and conserved tyrosines on human proteins

A combined list of human orthologs and paralogs for the respective Drosophila proteins was obtained using InParanoid (Version 6.0) hosted by SBC, HomoloGene hosted by NCBI and also using the eukaryotic ortholog list from Flybase.

Two different Blast searches (BLASTP hosted by NCBI and BLAST2 hosted by SIB blast network) were also made for individual whole protein sequence of tyrosine phosphorylated Drosophila proteins against the human protein database.

ClustalW2, a multiple sequence alignment program was used to align each Drosophila protein sequence against all the human protein matches from the blast searches to identify all the conserved tyrosines that are tyrosine phosphorylated in each of the 290 Drosophila proteins. The parameters used were: Protein Gap open penalty: 10.0; Gap Extension penalty: 0.2; Protein matrix: Gonnet; Protein ENDGAP: -1 and GAPDIST: 4.

## Supporting Information

Table S1 Overall classification of tyrosine phosphopeptides based on functional category of proteins. Summary of all the tyrosine phosphorylation sites that are specific to insulin RTK, EGF RTK and sites common to starved control cells/insulin/EGF treated cells. The conserved position of tyrosine on respective human ortholog and paralog are given for each phosphorylated site on Drosophila proteins. The Swiss-Prot protein ID for each human ortholog and paralog is also provided.
(0.58 MB XLS)

Table S2 Human orthologs and paralogs with conserved Y residues that are phosphorylated in Drosophila proteins. Summary of all the conserved tyrosines on human proteins. The details of each conserved tyrosine which includes the protein domain of tyrosine location, function, subcellular location, tissue specificity and information on disease phenotypes are given.
(0.36 MB XLS)

Table S3 Conserved phosphorylation sites on human proteins involved in various diseaeses. Summary of the list of putative tyrosine phosphorylation sites on various human proteins that are involved in the development of various cancers, leukemias, neurodegenerative diseases and various genetic syndromes/disorders.
(0.15 MB DOC)
