# Supplementary material for: Receptor Tyrosine Kinase (RTK) Mediated Tyrosine Phosphor-Proteome from Drosophila S2 (ErbB1) Cells Reveals Novel Signaling Networks
Source: PLoS One. 2008 Aug 6;3(8):e2877. doi: 10.1371/journal.pone.0002877 (PMC2488400; doi:10.1371/journal.pone.0002877)
Supplement: Table S3 — Conserved phosphorylation sites on human proteins involved in various diseaeses. Summary of the list of putative tyrosine phosphorylation sites on various human proteins that are involved in the development of various cancers, leukemias, neurodegenerative diseases and various genetic syndromes/disorders. (0.15 MB DOC) [file pone.0002877.s003.doc]

Supplementary Table3: List of important human proteins with conserved pY sites involved in various diseases

| **Protein with putative pY event** | **Accession #** | **Location of pY** | **Disease** |
| --- | --- | --- | --- |
| **ABCB5:Y39** | Q2M3G0 | ABC transporter 1 domain | Expressed in several malignant tissues |
| **ABCB7:Y579**  **ABCB7:Y701** | O75027 | ABC transporter domain | Defect leads to sideroblastic anemia with ataxia (ASAT) |
| **ABCG1:Y84** | P45844 | Cytoplasmic domain | Over-expressed in macrophages from patients with Tangier disease |
| **ABCG2:Y44** | Q9UNQ0 | Unknown | Up-regulated in brain tumors. |
| **ADAMTS20:Y 1572** | P59510 | TSP type-1 13 | Over-expressed in several brain, colon and breast carcinomas. |
| **ANK1:Y 1712** | P16157 | 55 kDa regulatory domain | Defects in ANK1 are a cause of hereditary spherocytosis (HS) |
| **ANKRD17:Y1404** | O75179 | ANK 25 repeat | Target of enterovirus 71 which is the major etiological agent of HFMD (hand, foot and mouth disease) |
| **AT1A2:Y539** | P50993 | Cytoplasmic domain | Defects in ATP1A2 are the cause of familial hemiplegic migraine 2 (FHM2),alternating hemiplegia of childhood (AHC) |
| **ATP1A3:Y532** | P13637 | Cytoplasmic domain | Defects in ATP1A3 are the cause of dystonia-12 (DYT12) also known as rapid-onset dystonia parkinsonism (RDP) |
| **BIRC6:Y4102**  **BIRC6:Y4130** | Q9NR09 | Unknown | Expressed in brain cancer cells. |
| **BRD4:Y670**  **BRD4-NUT:Y1046** | O60885 | Unknown | Rare, aggressive, and lethal carcinoma arising in midline organs of young people |
| **CACNA1F:Y1769** | O60840 | Cytoplasmic domain | congenital stationary night blindness type 2 (CSNB2) |
| **CENPF:Y1390** | P49454 | Unknown | Interacts with retinoblastoma protein (RB), CENP-E and BUBR1 |
| **CNBP:Y99; Y120** | P62633 | Zincefinger motiff:CCHC-type 4 | Defects in CNBP are the cause of myotonic dystrophy 2 (DM2) also known as proximal myotonic myopathy (PROMM) |
| **CNGB3:Y469** | Q9NQW8 | Cytoplasmic domain | Defects in CNGB3 are a cause of achromatopsia type 3 (ACHM3) also known as Pingelapese blindness |
| **CNTN4:Y880; Y940** | Q8IWV2 | Fibronectin type-III 3,  Fibronectin type-III 4 | Defects in CNTN4 are a cause of 3p deletion syndrome (3PDS). Defects in CNTN4 are the cause of spinocerebellar ataxia type 16 (SCA16) |
| **DAG1:Y831: Y863:Y886** | Q14118/ | cytoplasmic domain of Beta-dystroglycan chain | Forms part of the dystrophin-associated protein complex (DAPC). Is a target for the entry of Mycobacter |
| **DCC: Y1420**  **DCC:Y681** | P43146 | Cytoplasmic domain  Fibronectin type-III 3 | Colorectal tumors that lost their capacity to differentiate into mucus producing cells uniformly lack DCC expression |
| **DMBT1:Y1772** | Q9UGM3 | CUB 1 domain | Defects in DMBT1 are the cause of glioma of the brain. May be considered as a candidate tumor suppressor gene for brain, lung, esophageal, gastric, and colorectal cancers |
| **DMD-Dp140bc:Y934** | NP_004014 | Unknown | Defects in DMD are the cause of Duchenne muscular dystrophy (DMD), Becker muscular dystrophy (BMD), dilated cardiomyopathy also known as X-linked dilated cardiomyopathy (XLCM) |
| **DYR1A:Y136** | Q13627 | Domain:Bipartite nuclear localization signal | Over-expressed 1.5-fold in fetal Down syndrome brain |
| **E41L3:Y542**  **E41L3:Y872** | Q9Y2J2 | Carboxyl-terminal | Critical growth regulator in the pathogenesis of meningiomas. |
| **EPHB2:Y584** | P29323 | Cytoplasmic domain | Tumor suppressor |
| **EPN3:Y172** | Q9H201 | Unknown | Detected in chronic wounds, basal cell carcinoma and ulcerative colitis |
| **ETV6:Y391** | P41212 | ETS domain | Defects in ETV6 are a cause of acute myelogenous leukemia (AML), chronic myelomonocytic leukemia (CMML), childhood acute lymphoblastic leukemia (ALL), pre-B acute myeloid leukemia, myelodysplastic syndrome (MDS) with basophilia, acute eosinophilic leukemia |
| **FGFR1:Y463** | P11362 | Cytoplasmic domain | Pfeiffer syndrome, hypogonadotropic hypogonadism,Kallmann syndrome type 2, osteoglophonic dysplasia, metopic craniosynostosis, cell leukemia lymphoma, stem cell myeloproliferative disorder |
| **FGFR2:Y782** | P21802 | Cytoplasmic domain | Crouzon syndrome, Jackson-Weiss syndrome, Apert syndrome, Pfeiffer syndrome, Beare-Stevenson cutis gyrata syndrome, familial scaphocephaly syndrome, lacrimo-auriculo-dento-digital syndrome, Antley-Bixler syndrome |
| **Fibulin6:Y5045**  **Fibulin6:Y5296** | Q96RW7 | Nidogen G2 beta-barrel domain | Defects in HMCN1 are a cause of age-related macular degeneration type 1 (ARMD1) |
| **FLVC1:Y558** | Q9Y5Y0 | Unknown | May be required to protect developing erythroid cells from heme toxicity |
| **FOG1:Y591** | Q8IX07 | Zinc finger C2HC-type 2 | Tetralogy of Fallot (TOF), the most common cyanotic conotruncal heart defect (CTD), required for normal diaphragm and lung development |
| **FUS:Y208** | P35637 | Gly-rich domain | A chromosomal aberration involving FUS is a cause of a form of malignant myxoid liposarcoma, A chromosomal aberration involving FUS is a cause of acute myeloid leukemia (AML) |
| **GAS7:Y31** | O60861 | WW domain | A chromosomal aberration involving GAS7 is a cause of acute myeloid leukemia |
| **HACE1:Y677** | Q8IYU2 | HECT domain | Down-regulated in sporadic Wilms tumor |
| **HMCN1:Y 2861**  **HMCN1:Y3053** | Q96RW7 | Ig-like C2-type 26  between Ig-like C2-type 28 and 29 | Defects in HMCN1 are a cause of age-related macular degeneration type 1 (ARMD1) |
| **INVS:Y566** | Q9Y283 | IQ 1 domain | Defects in INVS are the cause of nephronophthisis 2 (NPHP2) |
| **IRS1:Y820** | P35568 | Unknown | Polymorphisms in IRS1 may be involved in the etiology of non-insulin-dependent diabetes mellitus (NIDDM) |
| **ITGA5:Y328** | P08648 | FG-GAP 5 repeat | In case of HIV-1 infection, the interaction with extracellular viral Tat protein seems to enhance angiogenesis in Kaposi's sarcoma lesion |
| **K0319:Y995** | Q5VV43 | Cytoplasmic domain | Defects in KIAA0319 may be a cause of susceptibility to dyslexia (DYX2) also called reading disability. |
| **L1CAM1:Y1070/N-CAML1:Y1075** | P32004 | Fibronectin type-III 5,Extracellular | Defects in L1CAM are the cause of corpus callosum hypoplasia, psychomotor retardation, adducted thumbs, spastic paraparesis, and hydrocephalus, hydrocephalus due to stenosis of the aqueduct of Sylvius,mental retardation, aphasia, |
| **LAMC2:Y1185** | Q13753 | Coiled-coil region | Defects in LAMC2 are a cause of junctional epidermolysis bullosa gravis (JEB) also known as junctional epidermolysis bullosa Herlitz-Pearson type |
| **LRP5:Y1426** | O75197 | Cytoplasmic domain | Defects in LRP5 are a cause of autosomal dominant and autosomal recessive familial exudative vitreoretinopathy (FEVR), involutional osteoporosis, osteoporosis pseudoglioma syndrome (OPPG), high bone mass trait (HBM), endosteal hyperostosis, van Buchem disease type 2 (VBCH2) |
| **LRP6:Y1541**  **LRP6:Y1562** | O75581 | Cytoplasmic domain | Defects in LRP6 are the cause of autosomal dominant coronary artery disease type 2 (ADCAD2) |
| **MDR1:Y1267** | P08183 | ABC transporter 2 domain | responsible for decreased drug accumulation in multidrug-resistant cells |
| **MERTK: Y520** | Q12866 | Transmembrane domain | Defects in MERTK are a cause of retinitis pigmentosa |
| **MFHA1:Y788** | Q9Y4C4 | Unknown | Overexpressed in malignant fibrous histiocytomas |
| **MLL2:Y2299/MLL2:Y2024** | O14686 | proline rich region | This gene mapped to a chromosomal region involved in duplications and translocations associated with cancer |
| **MOT8:Y198**  **MOT8:Y201**  **MOT8:Y280** | P36021 | Extracellular domain  Transmembrane domain  Cytoplasmic domain | Defects in SLC16A2 are the cause of monocarboxylate transporter 8 deficiency (MCT8 deficiency). Highly expressed in liver and heart |
| **MUSK:Y776** | O15146 | Protein kinase domain | Defects in MUSK is a cause of autosomal recessive congenital myasthenic syndrome (CMS) |
| **MXRA5:Y2709**  **MXRA5:Y2717** | Q9NR99 | Ig-like C2-type 11 | Over-expressed in arthritic tissues. Expression is reduced from young to old but increased from old to centenarians. |
| **MYH3:Y757** | P11055 | Actin binding region | Defects in MYH3 are the cause of distal arthrogryposis type 2A (DA2A) also known as Freeman-Sheldon syndrome (FSS). Also defects lead to distal arthrogryposis type 2B (DA2B) also known as Sheldon-Hall syndrome (SHS) |
| **MYO9B:Y2112** | Q13459 | Unknown | Genetic variation in MYO9B is the cause of susceptibility to celiac disease 4 (CELIAC4) |
| **MYST4:Y338** | Q8WYB5 | Unknown | A chromosomal aberration involving MYST4 may be a cause for acute myeloid leukemias. |
| **NDKB:Y67** | P22392 | Domain: Interaction with AKAP13 | This protein is found in reduced amount in tumor cells of high metastatic potential. |
| **NEBL:Y688** | O76041 | Nebulin 19 domain | Defects in NEBL are associated with non-familial idiopathic dilated cardiomyopathy (IDC) |
| **NOVA2:Y330** | Q9UNW9 | Alanine and glycine rich region | Autoantigen in the paraneoplastic opsoclonus myoclonus ataxia (POMA), a paraneoplastic neurological syndrome/disorder (PNS/D) associated with breast cancer, fallopian cancer, |
| **NPHN:Y 977** | O60500 | Fibronectin type-III domain | Defects in NPHS1 are the cause of congenital nephrotic syndrome of the Finnish type (NPHS1 or CNF) |
| **NUT:Y332** | Q86Y26 |  | A chromosomal aberration involving BRD4 is found in a rare, aggressive, and lethal carcinoma arising in midline organs of young people |
| **PCD15:Y1900** | Q96QU1 | Cytoplasmic domain | Defects in PCDH15 are the cause of Usher syndrome type 1F (USH1F), non-syndromic sensorineural deafness autosomal recessive type 23 (DFNB23) |
| **PGBM:Y3703** | P98160 | Laminin G-like 1 domain | Defects in HSPG2 are the cause of Schwartz-Jampel syndrome (SJS1), Defects in HSPG2 are the cause of dyssegmental dysplasia Silverman-Handmaker type (DDSH) |
| **PGFRB:970** | P09619 | Cytoplasmic domain | chronic myelomonocytic leukemia |
| **PMS1:Y631**  **PMS1:Y788** | P54277 | HMG box domain  Unknown | Defects in PMS1 are the cause of hereditary non-polyposis colorectal cancer type 3 (HNPCC3) also called Lynch syndrome |
| **PRG4:Y1290** | Q92954 | C terminal region | Defects in PRG4 are the cause of camptodactyly-arthropathy-coxa vara-pericarditis syndrome (CACP) also called Jacobs syndrome |
| **PROM1:Y828** | O43490 | Cytoplasmic domain | Selectively expressed on CD34 hematopoietic stem and progenitor cells in adult and fetal bone marrow, fetal liver, cord blood and adult peripheral blood. Defects in PROM1 are the cause of an autosomal recessive form of retinal degeneration. |
| **RAC2:Y64** | P15153 | Unknown | Defects in RAC2 are the cause of neutrophil immunodeficiency syndrome |
| **RASA1:Y952** | P20936 | Unknown | Mutations in the SH2 domain of RASA seem to be oncogenic and cause basal cell carcinomas, Defects in RASA1 are a cause of Parkes Weber syndrome (PKWS) |
| **RASH:Y157** | P01112 | Unknown | Defects in HRAS are the cause of Costello syndrome also known as faciocutaneoskeletal syndrome, bladder cancer, quamous cell carcinoma (OSCC). |
| **RASK:Y157** | P01116 | Unknown | Defects in KRAS are a cause of acute myelogenous leukemia (AML), juvenile myelomonocytic leukemia (JMML), Noonan syndrome 3 (NS3), cardiofaciocutaneous syndrome (CFC syndrome), KRAS mutations are involved in cancer development. |
| **RASN:Y157** | P01111 | Unknown | Defects in NRAS are a cause of juvenile myelomonocytic leukemia (JMML). Mutations which change AA 12, 13 or 61 activate the potential of Ras to transform cultured cells and are implicated in a variety of human tumors. |
| **REG1A:Y48**  **REG1A:Y49** | P05451 | C-type lectin domain on lithostathine 1 alpha chain | Alzheimer disease and Down syndrome patients show enhanced expression of PSP-related transcripts and intra-neuronal accumulation of PSP-like proteins in their brains |
| **REP1:Y438** | P24386 | Unknown | Defects in CHM are the cause of choroideremia also known as tapetochoroidal dystrophy (TCD) |
| **REP2:Y440** | P26374 | Unknown | Substitutes for REP-1 thereby preventing widespread tissue abnormalities in patients with choroideremia who lack REP-1. |
| **RHG26:Y371** | Q9UNA1 | Rho-GAP domain | Defects in ARHGAP26 are a cause of juvenile myelomonocytic leukemia (JMML) |
| **ROBO2:Y985** | Q9HCK4 | Cytoplasmic domain | Defects in ROBO2 are the cause of vesico-ureteral reflux type 2 (VUR2), A chromosomal aberration involving ROBO2 is a cause of multiple congenital abnormalities, including severe bilateral VUR with ureterovesical junction defects. |
| **S12A6:Y714** | Q9UHW9 | Transmembrane | Defects in SLC12A6 are a cause of agenesis of the corpus callosum with peripheral neuropathy (ACCPN) |
| **S12A8:Y441** | A0AV02 | Unknown | Defects in SLC12A8 may be a cause of susceptibility to psoriasis vulgaris (PSORS5) |
| **S19A1:Y149** | P41440 | Cytoplasmic domain | Uptake of folate in human placental choriocarcinoma cells |
| **S19A2:Y154** | O60779 | Cytoplasmic domain | Defects in SLC19A2 are the cause of thiamine-responsive megaloblastic anemia syndrome (TRMA) also known as Rogers syndrome |
| **S19A3:Y135** | Q9BZV2 | Cytoplasmic domain | Defects in SLC19A3 are the cause of biotin-responsive basal ganglia disease (BBGD) |
| **S38A5:Y19** | Q8WUX1 | Unknown | A chromosomal aberration involving SLC38A5 is found in 3 brothers with non-syndromic mental retardation. |
| **SLC22A3:Y49** | O75751 | Transmembrane | Mediates potential-dependent transport of a variety of organic cations. May play a significant role in the disposition of cationic neurotoxins and neurotransmitters in the brain |
| **SLC39A2:Y104** | Q9NP94 | Transmembrane | May be important in contact inhibition of normal epithelial cells and loss of its expression may play a role in tumorigenesis. |
| **SNX3:Y22** | O60493 | Unknown | A chromosomal aberration disrupting SNX3 may be a cause of syndromic microphthalmia type 8 (MCOPS8) |
| **SOAT1:Y429;Y431** | P35610 | Unknown | Accumulation of insoluble cholesterol esters in macrophages and smooth muscle is a characteristic feature of early lesions of atherosclerotic plaque |
| **SPAST:Y149** | Q9UBP0 | MIT domain | Defects in SPAST are the cause of spastic paraplegia type 4 (SPG4) |
| **SPDEF:Y302** | O95238 | ETS domain | Expressed in a very restricted set of primarily hormone-regulated epithelial tissues with particularly high expression in the prostate gland. |
| **SPTA1:Y986** | P02549 | SH3 domain | Defects in SPTA1 are the cause of elliptocytosis 2 (EL2), hereditary pyropoikilocytosis (HPP), spherocytosis type III (SPH3) |
| **STAT5B:Y725** | P51692 | Unknown | Defects in STAT5B are the cause of Laron type dwarfism II (LTD2) also known as Laron syndrome type II or Laron syndrome due to a post-receptor defect |
| **TAB3:Y501** | Q8N5C8 | Poly-serine rich region | Constitutively overexpressed in certain tumor tissues. Isoform 1 is a major transcript while Isoform2 is minor |
| **TBX1C:Y418**  **TBX1C:Y423**  **TBX1C:Y490** | O43435-3 | Unknown | Haploinsufficiency of the TBX1 gene is responsible for most of the physical malformations present in DiGeorge syndrome (DGS) and velocardiofacial syndrome (VCFS), conotruncal heart malformations (CTHM), Shprintzen syndrome |
| **TNKS2:Y569** | Q9H2K2 | ANK 11 repeat | Highly expressed in mammary gland, breast and breast carcinoma, |
| **TPM1:Y162** | P09493 | Unknown | Defects in TPM1 are the cause of familial hypertrophic cardiomyopathy type 3 (CMH3) |
| **TPM2:Y162** | P07951 | Unknown | Defects in TPM2 are the cause of nemaline myopathy type 4 (NEM4), distal arthrogryposis type 1 (DA1) |
| **TPM3:Y162** | P06753 | Unknown | Defects in TPM3 are a cause of nemaline myopathy type 1 (NEM1),thyroid papillary carcinoma (PACT) |
| **TRI33:Y336** | Q9UPN9 | region necessary for oligomerization | A chromosomal aberration involving TRIM33 is a cause of thyroid papillary carcinoma (PACT) |
| **UBR1:Y821, Y836, Y838** | Q8IWV7 | Unknown | Defects in UBR1 are a cause of Johanson-Blizzard syndrome (JBS), |
| **UNC5C:Y449** | O95185 | Cytoplasmic domain | Down-regulated in multiple cancers including colorectal, breast, ovary, uterus, stomach, lung, or kidney cancers |
| **VEGFR3:1063** | P35916 | Cytoplasmic domain | Defects in FLT4 are the cause of hereditary lymphedema, Defects in FLT4 are found in juvenile hemangioma |
| **VPP4:Y198** | Q9HBG4 | Extracellular | Defects in ATP6V0A4 are the cause of distal renal tubular acidosis with preserved hearing (RTADR) |
| **WNK1:Y545** | Q9H4A3 | Unknown | Defects in WNK1 are a cause of pseudo-hypoaldosteronism type II (PHAII) |
| **WWOX:Y34** | Q9NZC7 | WW 1 domain | Defects in WWOX may be involved in several cancer types. The gene spans the second most common chromosomal fragile site (FRA16D) which is frequently altered in cancers. |
